# British Institutions for the Care of the Inebriate.—IX

**Published:** 1904-04-09

**Authors:** 


### BRITISH INSTITUTIONS FOR THE CARE OF THE INEBRIATE.—IX.

By Our Own Medical Commissioner.

(*Continued from page* 13.)

### NORTHLANDS RETREAT, WANDSWORTH, LONDON.

In dealing with a morbid state such as inebriety, occurring as it does in both sexes, at all ages, and in every rank of society, much variety is necessary in the form of institution selected as a retreat. In some instances, where monetary considerations do not prevail, a country residence with extensive grounds and ample opportunities for an unrestricted outdoor life may be available, but for many with limited means, or dependent on the generosity of friends, an institution in or near a busy centre is frequently necessary.

"Northlands" is a retreat belonging to the latter class. It is conveniently situated, and consists of two old-fashioned houses in a terrace, with a shrubbery and carriage-drive in front. The address is 10–12 North Street, Wandsworth, S.W. The Misses Round are the resident licensees; their brother, Mr. John Round, L.R.C.P., L.R.C.S., is the medical licensee; and Dr. W. G. Dickenson is the visiting physician. "Northlands" was licensed in 1898 under the Inebriates Act, and is intended for a limited number of female cases, who are strongly recommended to seek admission under legislative provisions, although any lady may enter the Retreat as a private patient. The house, although lacking in the conveniences of modern construction and presenting much of an old-world air, has a comfortable and homely appearance. There is a morning-room, a dining-room, two drawing-rooms, and a number of bedrooms. Accommodation is provided for 18 patients.

Although the house is hemmed in by neighbouring building, there is a pleasant old-fashioned garden, with small lawn, and the back of the house has a venerable aspect with its training vine, clinging ivy, and drooping wisteria.

As far as we could judge in the course of our inspection the Home is conducted in accordance with rational views. Dr. W. G. Dickenson examines every case on admission and visits regularly once a week, and generally directs treatment.

Total abstinence from alcohol is strictly enforced. The licensees exercise a constant supervision, and accompany their charges on shopping excursions and visits to places of interest, and have all their meals and spend their evenings with the patients. During the summer months excursions are made to the numerous places of interest in the district, and in winter one of the principals accompanies any patients who may care to go to concerts and other entertainments. The neighbourhood is healthy, and many interesting walks are available. Putney Heath and Wandsworth, Clapham, and Wimbledon Commons are all quite near; and Hyde Park, Kensington, Richmond Park, Kew Gardens, etc., are easily reached by omnibus or tram.

All the servants are abstainers. No working patients are received.

The shortest time for which an inebriate is received is six months. The six months' patients are not allowed to go out alone. If a year's patient has done well, she is permitted to go for walks by herself when she has completed eleven months' residence. Patients frequently enter for a year, and remain for a further period of six or twelve months with their liberty. In only one instance has a patient been known to betray the trust in being allowed to have her liberty.

A large percentage of patients who have remained in the Home for a year are now said to be doing well in their own homes.

The terms are from one to two and a half guineas a week, according to bedroom selected. Laundry expenses are extra. Patients are received for short or long periods, or permanently.

The following "regulations and orders" are enforced at "Northlands," and go far to indicate the manner and methods of the institution:

1. The applicant on becoming an inmate of the Northlands Retreat for Inebriates will be required to conform to the regulations of the institution.
2. The admission paper must be filled up and signed by the person responsible for payment before the applicant enters the institution.
3. All letters written by the patients must be given to the principals, who will post them, unless they shall have good reason to know that they are written for the purpose of obtaining stimulants and drugs.
4. Patients may open their own letters or parcels if they contain neither stimulants nor drugs.
5. Patients may not enter any bedroom except their own.
6. Patients are not permitted to go out alone except in the garden.
7. Patients must be responsible for their books, papers, work, etc., if left in the sitting-rooms.
8. Patients may not have more than 2s. 6d. a week pocket-money without permission of the principals, and if they try to buy stimulants or drugs the pocket-money will be suppressed.
9. Patients must neither give, lend, nor borrow money.
10. Visitors are not allowed to bring stimulants or drugs into the retreat.
11. A patient may not take any stimulant or drug without special written authority from the medical attendant of the retreat.
12. Patients may go to see their friends, but the friends must fetch and bring them back, and patients must not be absent even for one night without due permission.
13. Patients may go to concerts, etc., with an attendant.
14. All patients are required to attend daily prayers and Church services on Sundays, except Roman Catholics or Jewesses.
15. Punctuality in respect to the hours of meals and other household arrangements to be strictly observed.
16. Suitable and sufficient clothing must be provided by the friends of each patient during her residence at the retreat; patients must find their own table-napkins and chamber-towels.

The daily routine is as follows:

Rise, 7.45 A.M.; breakfast, 9 A.M.; dinner, 1.30 P.M; tea, 5 P.M.; supper, 9 P.M.

There are morning prayers at 8.45 A.M., and evening prayers at 9.30 P.M.

